# Arbeitszeit bei Ärzt:innen und Auswirkungen auf Gesundheit, Zufriedenheit und Gesundheitsversorgung

**DOI:** 10.1007/s40664-023-00503-2

**Published:** 2023-04-28

**Authors:** F. U. Jung, M. Luppa, S. G. Riedel-Heller

**Affiliations:** Institut für Sozialmedizin, Arbeitsmedizin und Public Health (ISAP), Medizinische Fakultät, Universität Leipzig, Ph.-Rosenthal-Str. 55, 04103 Leipzig, Deutschland

**Keywords:** Arbeitszeit, Arbeitsbelastung, Arbeitszufriedenheit, Gesundheitsversorgung, Ärzteschaft, Working hours, Workload, Job satisfaction, Health care, Medical profession

## Abstract

Veränderungen der Arbeitswelt hinsichtlich innovativer Arbeitszeitmodelle erreichen zunehmend auch die Patient:innenversorgung, so steigt beispielsweise die Zahl der in Teilzeit arbeitenden Ärzt:innen kontinuierlich an. Gleichzeitig führt eine allgemeine Zunahme chronischer Erkrankungen und multimorbider Leiden, als auch der steigende ärztliche Personalmangel zu mehr Arbeitsbelastung und Unzufriedenheit. Die vorliegende Kurzübersicht fasst die aktuelle Studienlage hinsichtlich der Arbeitszeit von Ärzt:innen und damit verbundenen Konsequenzen zusammen und gibt einen ersten Überblick zu möglichen Lösungsansätzen.

## Abstract (en)

Changes in the working environment with respect to innovative working time models are also increasingly affecting patient care. The number of physicians working part-time, for example, is continuously rising. At the same time, a general increase in chronic diseases and multimorbid conditions as well as the growing shortage of medical staff, leads to more workload and dissatisfaction among this profession. This short overview summarizes the current study situation and associated consequences regarding working hours of physicians and gives a first explorative overview of possible solutions.

## Hintergrund

Die Arbeitsbelastung der Ärzt:innen ist in den vergangenen Jahren aufgrund der erhöhten Prävalenz chronischer Erkrankungen, der zunehmenden Bevölkerungsalterung, und einem damit verbundenen erhöhten Pflegebedarf, sowie wirtschaftlichen Herausforderungen und Personalmangel enorm gestiegen [53, 67]. Die Situation hat sich während der COVID-19-Pandemie noch einmal verschärft und einen Diskurs zum (politischen) Handlungsbedarf in Gang gesetzt [39].

Die Arbeitszeit von Ärzt:innen beinhaltet zusätzlich zur normalen täglichen Arbeitszeit häufig auch Bereitschaftsdienste, Nacht- und Schichtdienste sowie (regelmäßige) Überstunden [62]. Eine wichtige gesellschaftliche und gesundheitspolitische Frage ist, welchen Einfluss arbeitszeitbedingte Belastungen und verlängerte Dienstzeiten auf Gesundheit, Wohlbefinden und Zufriedenheit des ärztlichen Personals und – damit verbunden – auch auf das Versorgungssystem in seiner Gesamtheit haben.

Ziel der hier vorliegenden Kurzübersicht ist daher eine Bestandsaufnahme zu ärztlicher Arbeitszeit. Es folgt zunächst eine Übersicht zu rechtlichen Vorgaben und durchschnittlichen Arbeitszeiten, im Weiteren werden Konsequenzen hinsichtlich Arbeitsbelastung, Ärztegesundheit und Gesundheitsversorgung beschrieben.

## Arbeitszeit – welche Vorgaben gibt es und wie sieht die Realität aus?

### Rechtliche Vorgaben

In der Vergangenheit gab es bereits mehrfach gesundheitspolitische Debatten mit dem Ziel, die Arbeitsbedingungen – vor allem im Krankenhaus – zu verbessern. Im Zuge eines Urteils des Europäischen Gerichtshofes (EuGH RS C‑151/2 2003, EuGH, Rs. C‑303/98), nach dem Bereitschaftsdienste der Ärzt:innen nicht mehr als Erholungszeit, sondern als Arbeitszeit einzuordnen sind, gab es zum 1. Januar 2004 in Deutschland eine Änderung des Arbeitszeitgesetzes [42, S. 363]. Diese Änderung hatte das Ziel, die Arbeitsbelastung im ärztlichen Dienst zu verringern, die allgemeine Zufriedenheit der Krankenhausärzt:innen zu erhöhen und gleichzeitig die Patient:innenversorgung, insbesondere langfristig, zu optimieren.

Hinsichtlich der Ruhepausen gibt es ebenfalls im Gesetz verankerte Vorgaben. So müssen gemäß § 4 Arbeitszeitgesetz bei einer Arbeitszeit von sechs bis neun Stunden pro Tag mindestens 30 min Ruhepause eingehalten werden. Wer darüber hinaus arbeitet, dem stehen mindestens 45 min Pausenzeit zu. Die Lage der Ruhepausen muss dabei im Rahmen der Dienstplanung bzw. vor Antreten des Dienstes festgelegt werden (Landesarbeitsgericht Köln, Az.: 3 Sa 49/12). Am 7. März 2020 einigten sich Marburger Bund und Tarifgemeinschaft deutscher Länder (TdL) auf weitere Kernpunkte einer Tarifeinigung für Mitglieder im Geltungsbereich des TV-Ärzt:innen. Die Punkte umfassen u. a. eine verpflichtende Arbeitszeiterfassung durch Arbeitgeber, eine Begrenzung der Bereitschafts- und Wochenenddienste sowie die Pflicht zur rechtzeitigen Dienstplanerstellung. Betrachtet man die wöchentliche Arbeitszeit der Ärzteschaft, gilt es zwischen Subgruppen von Mediziner:innen zu unterscheiden, da es beispielsweise in Abhängigkeit vom medizinischen Setting (z. B. Krankenhaus, Medizinische Versorgungszentren, Niederlassung) und von der Fachrichtung zu Unterschieden kommen kann [9].

### Arbeitszeiten im Krankenhaus

Da vor allem Krankenhäuser rund um die Uhr an sieben Tagen in der Woche in Betrieb sind, um spezialisierte Gesundheitsversorgung sicherzustellen, ist die Arbeitszeit bei Krankenhausärzt:innen häufig von Wochenendarbeit, Nacht- und Schichtdiensten geprägt. Etwa 70 % der Krankenhausärzt:innen haben zusätzlich zu ihrer regulären Arbeitszeit Bereitschaftsdienste oder beraten außerhalb des Dienstes (Hausdienst; [57, 60]). In früheren Studien hat die Mehrheit der Ärzteschaft mindestens vier Bereitschaftsdienste pro Monat angegeben [34, 57, 76]. In einer deutschen Befragung von knapp 2000 Krankenhausärzt:innen gaben etwa 15 % an, mindestens neun Bereitschaftsdienste pro Monat am Arbeitsplatz absolviert zu haben [57]. Krankenhausärzt:innen berichten insgesamt von langen Wochenarbeitszeiten oft über 50 h [20, 58, 59, 68] und in einer Studie sogar deutlich über 60 h [15].

### Arbeitszeiten in ambulanten Bereichen

Im Rahmen der Erhebungswelle 2020 des ZI-Praxis-Panels [79] ergab sich eine durchschnittliche Wochenarbeitszeit von etwa 46 h bei niedergelassenen Praxisinhaber:innen (Vertragsärzt:innen und Vertragspsychotherapeut:innen). Ärzt:innen aus den Fachrichtungen Innere Medizin, Urologie, Neurologie und Nervenheilkunde bzw. Neurologie und Psychiatrie gaben an, 50 h und mehr zu arbeiten und haben damit die höchsten Wochenarbeitsstunden. Ärzt:innen in ländlichen Regionen arbeiten durchschnittlich mehr als ihre städtischen Kolleg:innen (48 h vs. 44 h).

Ein Trend, der sich bereits seit einigen Jahren in verschiedenen Berufsfeldern beobachten lässt, ist der Wunsch nach Teilzeitarbeit. Auch in der Medizin spielt dies zunehmend eine Rolle und betrifft Mediziner:innen unabhängig von Qualifikationsstufe oder Karriereplänen [27, 37, 40, 65, 69]. Dies spiegeln auch die Ergebnisse des Praxis-Panels wider. Betrachtet man Inhaber:innen und Angestellte zusammen, lag die wöchentliche Arbeitszeit nur bei 35 h. Vergleicht man die Daten früherer Erhebungen des ZI-Praxis-Panels, bestätigt sich die Tendenz zur Arbeitszeitreduktion. So lag die durchschnittliche Wochenarbeitszeit 2014 im Durchschnitt bei 42 h, im Jahr 2015 waren es 41 h und im Jahr 2017 waren es 39 h [18–20]. Gründe für eine Reduktion der Arbeitszeit oder für eine Teilzeittätigkeit sind vielfältig. Nationale und internationale Studien zeigen, dass einerseits Arbeitsbelastung, aber auch außerberufliche Interessen und eine allgemeine Zunahme der Bedeutung von Familie und Freizeit den Wunsch nach Arbeitszeitreduktion begünstigen können [8, 10, 27, 66].

## Arbeitszeitbezogene Auswirkungen und Konsequenzen

### Arbeitszeit und mentale Gesundheit

In der jüngeren Vergangenheit sind vereinzelt Beiträge vorzufinden, die sich mit den (gesundheitlichen) Auswirkungen von Arbeitszeitmodellen speziell im ärztlichen Dienst befassen. Aus anderen Berufsgruppen und Studien ist bereits bekannt, dass die psychische Gesundheit einer Person durch ständigen Arbeitsstress stark beeinträchtigt werden kann [74]. Lange Arbeitszeiten sind einer der wichtigsten Stressoren, die sich negativ auf die psychische Gesundheit auswirken, insbesondere in Bezug auf Angststörungen und Depressionen [17, 46, 71]. Eine Querschnittstudie mit 258 Assistenzärzt:innen zeigte, dass lange Arbeitszeiten (in dieser Studie definiert als eine wöchentliche Arbeitszeit von mehr als 64 h) mit einem signifikant erhöhten Risiko einhergeht, eine Angststörung und/oder eine Depression zu entwickeln [9]. Im Widerspruch dazu stehen Ergebnisse einer longitudinalen Studie, welche keinen direkten Zusammenhang zwischen der Anzahl der Arbeitsstunden und Depressivität bestätigen konnte [73]. Vielmehr hat sich hierbei gezeigt, dass bestimmte Arbeitszeitdimensionen in diesem Zusammenhang von großer Relevanz sind. So zeigte sich beispielsweise, dass der Zusammenhang zwischen freien Wochenenden und einem reduzierten Risiko, depressive Symptome zu entwickeln, als eine Proxy-Schätzung für den Zusammenhang von Überstunden mit inzidenten depressiven Symptomen angenommen werden könnte [77]. Die Studien weisen also daraufhin, dass weitere Faktoren, darunter Alter, familiäre Verpflichtungen, soziale Fähigkeiten, Zufriedenheit mit dem Fachgebiet und körperliche Aktivität ebenfalls berücksichtigt werden sollten. Welche Rolle dabei arbeitszeitbedingte Einflüsse auf den Schlaf spielen, wird nachfolgend näher beschrieben.

### Arbeitszeit, allgemeine Gesundheit und Schlaf

In einer Stichprobe mit 2845 Klinikärzt:innen konnte gezeigt werden, dass lange Arbeitsschichten, Bereitschaftsdienste und kurze Schichtintervalle mit dem Risiko kurzer Krankheitsausfälle von 1-3 Tagen bei Krankenhausärzt:innen in Zusammenhang stehen [55]. Eine weitere Studie untersuchte die Zusammenhänge zwischen Schlaf, Arbeitszeit und Bereitschaftsdienst von 728 Krankenhausärzt:innen. Häufige Nachtarbeit und lange wöchentliche Gesamtarbeitszeit waren dabei mit kurzer Schlafdauer verbunden. Sowohl die insgesamt langen Wochenarbeitszeiten als auch mehrere spezifische Merkmale der Bereitschaftsarbeitszeit (z. B. Anzahl der Dienste innerhalb einer Woche, Aufenthalt zu Hause oder im Krankenhaus) waren mit unzureichendem Schlaf verbunden. Obwohl fast die Hälfte der Ärzt:innen häufig Schlafstörungen hatte, waren in einer separaten Analyse dieser Studie nur häufige Nachtdienste mit Einschlafstörungen verbunden [31].

Zu den Mechanismen, die hierbei zugrunde liegen, gehören unzureichender Schlaf und fehlende Erholungszeiten sowie gestörte zirkadiane, biologische Rhythmen während der Nachtarbeit oder (über)langen Arbeitszeiten [1, 3, 30, 45, 62, 64, 76]. Basierend auf einer systematischen Literaturrecherche wurde hierzu ein umfassendes Modell vorgeschlagen, welches Erklärungen für den Zusammenhang zwischen atypischen Arbeitszeiten und Gesundheit [41] liefert. Nach diesem Modell weisen Faktoren, die die Arbeitszeit betreffen (beispielsweise Schichtdauer und Rotation) und Faktoren, die nicht auf die Arbeitszeit bezogen sind (d. h. Dauer der Erholungsperiode, Privatleben) synergistische Effekte auf physiologische Prozesse (d. h. zirkadiane Störungen) auf und haben kurz- sowie langfristig gesundheitliche Auswirkungen auf die Betroffenen. Auswirkungen von Schichtarbeit und Nachtdiensten wurden ebenfalls ausführlicher überprüft. Störungen des zirkadianen Zyklus sowie unzureichende Ruhezeiten können sowohl körperliche als auch psychische Gesundheitsprobleme hervorrufen [43]. Überstunden bzw. lange Arbeitszeiten beeinträchtigen ebenfalls die Gesundheit, zum Beispiel durch eine Erhöhung des Risikos für Herz-Kreislauf-Erkrankungen, Depressionen und somatische Beschwerden in verschiedenen Berufsgruppen, aber auch bei Ärzt:innen [5, 16, 54, 75]. Atypische Arbeitszeiten (z. B. Arbeiten an Wochenenden, Bereitschaftsdienste) können nicht nur die Work-Life-Balance beeinträchtigen und die Gesamtzufriedenheit reduzieren [2], sondern auch zu unregelmäßigem Schlaf, schlechteren Ernährungsgewohnheiten und Fettleibigkeit führen sowie Substanzkonsum begünstigen [78].

Ergebnisse aus ärztlichen Stichproben lassen vermuten, dass vor allem unzureichender Schlaf und fehlende Erholungszeiten aufgrund langer oder unregelmäßiger Arbeitszeiten mit negativen Konsequenzen für die Gesundheit und Zufriedenheit einhergehen, obwohl die Evidenz aufgrund methodischer Einschränkungen als nicht gesichert gilt [55]. Die meisten Studien zur Bereitschaftsarbeit basieren auf selbstberichteten Daten, die anfällig für Gedächtnisverzerrungen sind [50, 52], im Querschnittsdesign erhoben wurden [36] oder begrenzte Gruppen medizinischer Fachrichtungen beinhalten [14, 19, 36]. So gibt es beispielsweise mehr Evidenz aus Studien, welche Klinikärzt:innen in die Studie einschlossen. Evidenz, die sich auf Ärzt:innen in eigener Niederlassung oder angestellte Ärzt:innen im ambulanten Bereich stützt, ist derzeit kaum vorhanden. Gleichzeitig gilt die Datenlage hinsichtlich ärztlicher Arbeitszeit als unzureichend [6, 70].

### Arbeitszeit und Arbeitsbelastung

Psychische Gesundheitsprobleme im Allgemeinen können Konzentration, Arbeitsleistung und die Qualität der Versorgung beeinträchtigen und somit das Risiko medizinischer Fehler erhöhen [25]. Die empirischen Untersuchungen zur Arbeitszeitorganisation und Arbeitsbelastung weisen dabei weitestgehend übereinstimmend einen Zusammenhang nach [4, 24, 49, 56].

Demnach führt eine hohe Anzahl an Arbeitsstunden zu einer erhöhten Arbeitsbelastung der Ärzt:innen [4, 24]. Hohe Arbeitsbelastungen und arbeitsbezogener Stress können wiederum das Risiko für Burnout erhöhen [61, 80]. Die Prävalenz von Mediziner:innen mit Burnout wird für Deutschland auf 4–20 % geschätzt [7]. Es gib bereits Belege für eine hohe Korrelation zwischen Überstunden und Burnout bei Ärzt:innen [47]. Im Vergleich zu einer wöchentlichen Arbeitszeit von 40 h kann sich das Burnout-Risiko bei 60 Wochenstunden verdoppeln und bei mehr als 74 Wochenstunden sogar verdreifachen [35]. Im Gegensatz dazu scheint eine als besser wahrgenommene Arbeitszeitplanung sowie Autonomie und Flexibilität hinsichtlich der Arbeitszeitplanung die Arbeitsbelastung zu senken [28, 49]. Baldwin et al. [4] sowie Hutter et al. [24] zeigen außerdem auf, dass eine Beschränkung der wöchentlichen Höchstarbeitszeit die Arbeitszufriedenheit erhöht [4, 24]. Ärzt:innen haben unregelmäßige Arbeitszeiten, z. B. Arbeitszeiten mit Bereitschafts- oder Notdienst, unterschiedlichen Start- und Endzeiten sowie variierende Schichtlängen und Erholungszeiten [54, 59, 64]. Auch Bereitschaftsdienste sind mit einer Reihe negativer gesundheitlicher Folgen verbunden und korrelieren mit Arbeitsstress, Burnout, Arbeitsunfällen und krankheitsbedingten Fehlzeiten [13, 36, 38, 59].

### Auswirkungen auf die Patient:innenversorgung

Hinsichtlich der möglichen Konsequenzen auf die Patient:innenversorgung gibt es bereits eine Vielzahl von Belegen. So beeinflusst der Bereitschaftsdienst beispielsweise die Fluktuationsabsichten: Die höchsten Fluktuationsabsichten waren bei jenen Ärzt:innen zu verzeichnen, die sowohl Bereitschaftsdienst hatten als auch eine hohe Arbeitsbelastung erlebten [23].

Weitere Studien belegen einen Zusammenhang zwischen arbeitszeitbedingter Arbeitsbelastung und dem Wunsch, die Arbeitszeit zu reduzieren oder einen vorzeitigen Ruhestand in Erwägung zu ziehen. In einer Befragung unter sächsischen Hausärzt:innen zwischen 29 und 66 Jahren zeigte sich beispielsweise, dass 50 % der Befragten vorzeitig in den Ruhestand gehen wollen und diese Entscheidung u. a. mit der Zufriedenheit bezüglich der Arbeitszeit, aber auch mit der Arbeitsbelastung allgemein zusammenhängt [29].

Zu ähnlichen Ergebnissen kommt eine Längsschnittstudie, an welcher 16.681 Ärzt:innen verschiedener Fachrichtungen teilnahmen [32]. Hierbei konnte gezeigt werden, dass die Zufriedenheit der Ärzt:innen eng mit der Entscheidung, in den Ruhestand zu gehen und die Arbeitszeit zu reduzieren, zusammenhängt. Unzufriedene Ärzt:innen gingen 2‑ bis 3‑mal häufiger in den Ruhestand oder arbeiteten weniger als 20 h pro Woche als zufriedene Ärzt:innen. Arbeitszeit sollte also auch in diesem Kontext nicht als eine wichtige Determinante außer Acht gelassen werden. Allerdings weisen die Ergebnisse auch darauf hin, dass die Entscheidung zur Terminierung des Ruhestandes stark mit der Spezialisierung bzw. Fachrichtung zusammenhängt und dass die Entscheidung, weniger Stunden zu arbeiten, davon abhängt, ob die Person Praxisinhaber:in ist.

Unter dem Aspekt des Ärztemangels können jedoch ärztliche Entscheidungen für einen Austritt aus der Gesundheitsversorgung mit immensen Kosten für das Gesundheitssystem verbunden sein. Auch wenn es derzeit noch keine Daten aus Deutschland gibt, so weisen internationale Studien darauf hin, dass Fluktuation und verkürzte Arbeitszeiten bei Ärztinnen und Ärzten aufgrund von Arbeitsbelastung oder Überlastung beispielsweise in den USA jährlich Kosten in Höhe von etwa 4,6 Mrd. US-Dollar verursachen [18].

Aus bisherigen Studien ist auch bekannt, dass bestimmte Arbeitszeitdimensionen – beispielsweise Bereitschaftsdienste – negative Auswirkungen auf die Patientensicherheit haben können. Schichtlänge und damit einhergehende Ermüdungserscheinung sind wichtige Determinanten hinsichtlich Patientensicherheit und unerwünschter Behandlungsfehler [48, 72]. Eine umfangreiche Literaturrecherche dazu zeigt jedoch, dass Arbeitszeitbeschränkungen per se häufig nicht zu verbesserten Indikatoren für die Patientensicherheit führen [44]. Andere Faktoren, wie beispielsweise Anzahl oder Dauer der Erholungsphasen sollten ebenfalls berücksichtigt werden. Eine höhere Komplikationsrate bei chirurgischen Eingriffen nach Nachtdiensten wurde demnach beobachtet, wenn der Chirurg zwischen den Arbeitsschichten weniger als 6 h Ruhe hatte, verglichen mit 6 oder mehr Stunden Ruhe [63]. Gleichzeitig weist eine Studie daraufhin, dass arbeitsbedingte Schlafstörungen zu Erschöpfung und zu Behandlungsfehlern führen [72]. Die Autoren der Studie haben darauf aufbauend ein Modell entworfen, welches erklärt, weshalb eine reduzierte Aufmerksamkeitsfähigkeit zu einem Teufelskreis beitragen kann. Demnach kann Schlafentzug (durch übermäßige Arbeitszeiten) nicht nur die Arbeitsfähigkeit beeinträchtigen und Fehler hervorrufen, sondern auch zu einer erhöhten Arbeitszeit beitragen, beispielsweise, wenn Untersuchungen oder Behandlungen aufgrund reduzierter Arbeitsfähigkeit mehr Zeit in Anspruch nehmen oder erneut durchgeführt werden müssen.

## Diskussion und Perspektiven

Die vorliegende Kurzübersicht zur Arbeitszeit bei Ärzt:innen zeigt, dass die Untersuchung der Thematik für die Sicherung der Patientenversorgung unumgänglich ist. Studienergebnisse weisen darauf hin, dass die Thematik einem stetigen Wandel des Arztberufes, den Bedürfnissen und Wünschen dieser Berufsgruppe als auch den Veränderungen im Gesundheitswesen und der Gesellschaft unterliegt. Arbeitszeit kann sich demnach nicht nur auf die Gesundheit und Arbeitsfähigkeit der Ärzteschaft auswirken, sondern auch auf die Patientensicherheit.

Dabei soll der Wunsch nach Teilzeitarbeit oder Arbeitszeitreduktion nicht unbedingt als ursächlich für den voranschreitenden Ärzt:innenmangel gesehen werden, sondern vielmehr als Möglichkeit für Veränderung. Attraktive Arbeitszeiten können in Zeiten von Ärzt:innenmangel auch ein Mittel sein, um Fachkräfte anzuziehen.

Eine Steigerung der Attraktivität dieser Profession durch moderne und attraktive Arbeitszeiten ist ein wichtiger Schritt, um Abwanderung in Berufe außerhalb der Patientenversorgung sowie vorzeitigen Eintritt in den Ruhestand zu verhindern.

Trotz allem weisen derzeitige Studien darauf hin, dass es gerade in diesem Forschungsbereich eine Vielzahl von Lücken gibt, die geschlossen werden müssen. Es stellt sich beispielsweise die Frage, inwiefern die mit innovativen Arbeitszeitmodellen verbundenen Kosten niedriger ausfallen, als Kosten durch (arbeitszeitbedingte) Überlastung und Burnout. Gleichzeitig sollte die Etablierung innovativer Arbeitszeitmodelle in allen medizinischen Settings kritisch und umfangreich geprüft werden. Beispiele für Arbeitszeitmodelle können eine Reduktion der Arbeitszeit/Teilzeitarbeit oder Reduktion der Arbeitstage (z. B. 4‑Tage-Woche) sowie Wechselmodelle (Arbeitswochen gefolgt von arbeitsfreien Wochen) beinhalten. Hier stellt sich einerseits die Frage, inwiefern es durch diese Modelle zu Veränderungen bzw. Verbesserungen kommt, und andererseits, was dies für die Patientenversorgung langfristig bedeuten kann. Erste Ergebnisse einer Befragung mit 261 Klinikärzt:innen zeigen, dass neue, innovative Arbeitszeitmodelle die Arbeitsbelastung verringern und die Arbeitszufriedenheit der Ärzt:innen verbessern, sich die Möglichkeiten der Patientenversorgung allerdings nicht signifikant verändern [22].

Folgende Möglichkeiten wurden von Chang und Kollegen [12] vorgeschlagen, um dem Personalmangel entgegenzuwirken, Arbeitszeiten zu regulieren, Überstunden zu vermeiden und langfristig ärztliches Personal zu entlasten:

1. Miteinbezug von Medizinstudent:innen (d. h. frühzeitige Arbeit am Patienten),
2. Rekrutierung ausländischer Ärzt:innen,
3. Erhöhung der Anzahl der Mitarbeiter:innen, die einige der klinischen Aufgaben eines Arztes/einer Ärztin übernehmen können, z. B. speziell ausgebildete Arzthelfer:innen oder Physician Assistants (Bachelor-Studium),
4. Reduzierung der nichtklinischen Aufgaben der Ärzt:innen sowie Entbürokratisierung in Krankenhäusern und ambulanten Einrichtungen [12].

Eigene Arbeiten zu Arbeitszeit bei Ärztinnen und Ärzten weisen darauf hin, dass insbesondere Arbeitszeitdimensionen und Arbeitszeitflexibilität einen Einfluss auf Arbeitszufriedenheit, allgemeine Gesundheit und Burnout-Risiko haben und damit eine wichtige Stellschraube für die Verbesserung der Arbeitsbedingungen darstellen können [28, 29]. So konnte gezeigt werden, dass eine erhöhte Autonomie hinsichtlich der Arbeitszeit (z. B. hinsichtlich Arbeitsbeginn und Arbeitsende sowie Möglichkeit der Rücksichtnahme auf private Interessen innerhalb der Arbeitszeitplanung) mit weniger Arbeitsbelastung und Burnout einhergeht und die Arbeitszufriedenheit gesteigert werden kann. Zukünftige Studien sollten diese Zusammenhänge längsschnittlich untersuchen und gleichzeitig Möglichkeiten entwickeln, wie Selbstbestimmung hinsichtlich der Arbeitszeit im ärztlichen Arbeitsalltag gefördert werden kann, wie sich innovative Arbeitsmodelle umsetzen und etablieren lassen und welche Auswirkungen sich auf Gesundheit und Befinden als auch Einstellung zum Beruf und Entscheidungen hinsichtlich des Ruhestandes ergeben. Dies ist vor allem für Ärztinnen von Bedeutung, die Familie und Arztberuf miteinander vereinbaren wollen oder denen es aufgrund unflexibler Arbeitszeitmodelle verwehrt bleibt, einen Karriereweg innerhalb der Klinik einzuschlagen [11, 33]. In diesem Zusammenhang sollte auch die Möglichkeit einer (ambulanten) Weiterbildung in Teilzeit untersucht werden. Bisherige Studien weisen darauf hin, dass viele Ärzt:innen den Weg in eine ambulante Tätigkeit gehen, weil ihnen hier das Arbeiten in Teilzeit in Aussicht gestellt wird. Es stellt sich die Frage, inwiefern dies auch während der Facharztausbildung eine Rolle spielt und wie Ärzt:innen diese Möglichkeit wahrnehmen bzw. nutzen.

Im Rahmen der Thematik handelt es sich um einen Spagat zwischen den individuellen Bedürfnissen der Ärzteschaft (z. B. Familienplanung, Weiterbildung, Arbeit außerhalb der Patientenversorgung) und den Anforderungen des Gesundheitssystems. Gerade in einer älterwerdenden Bevölkerung, die von einer Zunahme chronischer Erkrankungen betroffen ist, ist es schwer, den Wunsch nach reduzierter Arbeitszeit zu realisieren. Zunehmende Arbeitsverdichtung in Krankenhäusern oder der bürokratische Aufwand in ambulanten Bereichen sind nur zwei Beispiele, die von Ärzt:innen häufig als Ursache für (vermeidbare) Arbeitsbelastung genannt werden [21, 51]. Wenn Ärzt:innen am Rande der Erschöpfung arbeiten oder gar von Präsentismus die Rede ist, kann jedoch keine optimale Gesundheitsversorgung garantiert werden. Gerade neuere Studien weisen darauf hin, dass es einen Zusammenhang zwischen Arbeitszeitreduktion und hoher Arbeitsbelastung gibt [26]. Wichtig ist es daher, gemeinsam nach einer Lösung zu suchen, die die Interessen aller Beteiligten berücksichtigt.

## Fazit für die Praxis

- Veränderungen der Arbeitswelt betreffen zunehmend auch das Gesundheitswesen. Autonomie und Selbstbestimmung sind dabei zwei wichtige Säulen.
- Gleichzeitig gilt es auf Bedürfnisse der Ärzteschaft einzugehen, Arbeitsstress und Belastungen langfristig zu reduzieren und eine effiziente und lückenlose Versorgung von Patient:innen zu gewährleisten.
- Studien weisen darauf hin, dass die Bedeutung der Arbeitszeit in der ärztlichen Profession eine wichtige Rolle einnimmt und es großen gesundheitspolitischen Handlungsbedarf gibt.
- Zukünftige Forschung sollte dazu beitragen, Möglichkeiten für innovative Arbeitszeitmodelle zu erproben und zu etablieren, um Medizinerinnen und Mediziner in ihrem Beruf zu stärken und gleichzeitig die Attraktivität hinsichtlich Berufswahl sowie Ausübung zu erhöhen.
